# Comparative analysis of bioactive compounds and antimicrobial activity in marine cyanobacteria

**DOI:** 10.1038/s41598-026-59496-6

**Published:** 2026-07-13

**Authors:** Reham Gamal, Ahmed Ismail, Nader Saad Elsayed, Ola Kh. Shalaby

**Affiliations:** 1 National Institute of Oceanography and Fisheries (NIOF), Cairo, Egypt; 2 Pharmacognosy Department, Faculty of Pharmacy, Fayoum University, Fayoum, Egypt; 3 Soil and Agricultural Chemistry Department, Faculty of Agriculture (Saba-Basha), Alexandria University, Alexandria, Egypt; 4 School of Civil Engineering, Southeast University, Nanjing, China

**Keywords:** Marine cyanobacteria, Antimicrobial, Phytochemical, GC-Mas, Biological techniques, Biotechnology, Environmental sciences, Microbiology

## Abstract

A comparative study of bioactive compounds in two marine cyanobacteria species, Oscillatoria acutissima and Oscillatoria simplicissima, grown in F/2 media was performed by estimation of many of bioactive compounds after 12th days, The greatest level of Phenolics and tannins was observed in *Oscillatoria acutissima*. Methanolic extracts of two species of cyanobacteria demonstrated diverse antimicrobial activity toward various human pathogenic microbial species which were associated with their bioactive components. Two species of *cyanobacteria* were observed to be the most active one in relation to all examined pathogenic microorganisms. The methanolic extracts of the algae were chemically distinguished by gas chromatography mass spectroscopy “GC–MS”. Based on the results obtained, the two species of marine cyanobacteria can be a renewable provider of beneficial bioactive complexes for health and nutritional products.

## Introduction

Cyanobacteria are gram-negative, photosynthetic prokaryotic organisms that have the ability to grow in a wide range of aquatic and terrestrial habitats^1^. Their structural diversity and capacity to produce a wide range of compounds such as pigments, vitamins, and enzymes make them pioneer species in most of the ecosystems. Additionally, cyanobacteria are known to be sources of a wide range of natural products, some of which are toxins, but have potential applications in the pharmaceutical industry^2–4^. Nostoc genus is a morphologically diverse and large group of phototrophic cyanobacteria that are located in various environments. The presence of both primary and secondary metabolites with varied bioactive properties in plants and cyanobacteria underscores their importance to phytopharmaceutical uses^5^. Cyanobacteria have antioxidant, anticancer and antiviral properties and find applications in a wide range of industries, such as agriculture, industry, medicine, biotechnology, and pharmaceuticals^6^. They also exhibit antimicrobial effects capable of inhibiting or destroying pathogenic microorganisms. These antimicrobial agents work by modifying or disrupting cytoplasmic membrane, disabling enzymes, and preventing the synthesis of proteins^7^. The antimicrobial effect differs according to the algal species and the kind of solvent used^8^. Moreover, cyanobacteria synthesize natural compounds that enhance their survival under diverse environmental stresses. These natural metabolites have been applied in disease management for decades, and cyanobacteria continue to offer promising sources for developing novel drugs against previously incurable diseases^9^. Recent progress in biotechnology has focused on enhancing the synthesis of valuable compounds in cyanobacteria for use across diverse industrial sectors^10^. Cyanobacteria are known to generate a range of bioactive metabolites, such as those with antitumor properties^11^, toxic compounds^12^, and enzyme inhibitors^13^. These metabolites serve multiple biological roles, including defense against predators, chemosensory signaling, and protection from light-induced damage. Because of their adaptive responses to antimicrobial agents, such bioactive substances have potential applications in nutraceuticals, pharmaceuticals, and industrial biotechnology, particularly in the development of cosmeceuticals^14^. This adaptive capacity underscores the importance of ongoing investigations into novel antimicrobial agents^15^. The current study focuses on analyzing the phytochemical composition of two cyanobacterial strains gathered from the Gulf of Aqaba along the Red Sea coast of Alexandria, Egypt, with the goal of identifying their chemical constituents for potential industrial and other practical uses.

## Materials and methods

### Algal Isolation

The study’s algae species were gathered from the Gulf of Aqaba on Alexandria’s Red Sea coast. The F/2 medium was used to culture the samples^16^. After that, the medium was autoclaved for 30 min at 120 °C. The culture was incubated at pH 8, 30 ± 1 °C, and 3000 lx of light intensity. The ideal conditions were maintained for the algae. Based on the available literature, the isolated strain was recognized morphologically^17^.

The marine cyanobacterial strains used in this study were isolated from environmental water samples. Monocultures of single species were successfully separated and established using the standard capillary micropipette washing technique under an optical microscope, followed by successive streak-plating onto F/2 agar plates to ensure unialgal purity before scale-up.

The study’s algae species were gathered from the coastal waters of Alexandria, Egypt. The two marine cyanobacteria species (*Oscillatoria acutissima* and *Oscillatoria simplicissima*) were cultured separately in 500 mL glass Erlenmeyer flasks containing a working volume of 300 mL of sterile F/2 medium. The medium was prepared using natural seawater filtered through 0.45 μm membranes and adjusted to a baseline marine salinity of 35‰. The medium was autoclaved for 30 min at 120 °C. Culturing was conducted in a controlled environmental incubator at a temperature of 25 ± 2 °C, under a light intensity of 3000 lx with an operating pH of 8. A strict 16:8 h light: dark diurnal photoperiod cycle was maintained. To ensure continuous gas exchange and prevent biomass sedimentation, the cultures were continuously agitated by bubbling with sterile-filtered air using an automated aeration system. Based on the available literature, the isolated strains were recognized morphologically.

### Measurements of algal growth

As per the procedure outlined by^18^. measuring the growth of algae using chlorophyll a. Centrifugation was used for 15 min at 5000 rpm in order to harvest. By comparing the absorbance at 663 and 645 nm in a 1 cm quartz cell to a blank of 80% hydrous acetone using a spectrophotometer, the pigment concentration in the filtered extract was calculated employing the subsequent formula:

Chlorophyll a = 12.7. E^663^-2.69. E^645^.

### Preparation of the algal extracts

In F/2 media, three microalgae were cultivated under aeration. In order to extract antimicrobial compounds, microalgae pellets were collected for development during the stationary stage, the culture was subjected to centrifugation, and the pellets were subjected to hot air drying (60 °C) until they reached a consistent weight. Each of the three microalgae’s half-gram dry biomass was extracted using ten millilitres of hexane, chloroform, ethanol, and methanol. According to^19^, every extract was kept at -4° C.

“In F/2 media, the microalgae were cultivated under continuous aeration. In order to extract antimicrobial compounds, microalgae pellets were collected during the stationary stage via centrifugation at 4000 rpm for 15 minutes. The collected algal pellets were washed with distilled water and subjected to hot air drying (60°C) until they reached a constant weight. For extraction, a half-gram of each microalga’s dry biomass was extracted using ten millilitres of absolute analytical-grade methanol under continuous shaking at 150 rpm for 48 hours at room temperature in the dark. The mixture was filtered using Whatman No. 1 filter paper, and the solvent was completely evaporated under reduced pressure using a rotary evaporator at 40°C to yield the crude methanolic extract. According to^19^, every extract was kept at -4°C.”

### Phytochemical characteristics

#### Extraction of secondary metabolites

Dried samples, 10 g, were placed within Soxhlet equipment and obtained with 100 ml methanol for 8 h, subsequently the filtrate (crude extracts) was gathered.

#### Formulation of methanolic algae extracts

Approximately 1 g of every dried algal biomass sample was individually blended in methanol and subjected to sonication for 20 min employing an ultrasonic micro tip probe (400 W, ULTRASONIC Get 750). The mixtures were subsequently subjected to centrifugation for 10 min at 4500 rpm. The resulting supernatants were gathered independently, while the remaining pellets were subjected to extraction again two times following the same procedure. All collected supernatants were combined and refrigerated for subsequent analysis. Some portions of the supernatants underwent evaporation until dry at 40 °C employing a rotary evaporator, and the resulting dried samples were transferred into marked sterile vials and preserved at − 20 °C in a deep freezer for antimicrobial evaluation^20^.

#### Qualitative analysis

Phenolics, phytosterols, triterpenes, saponins, tannins, flavonoids, anthraquinones, coumarins and cardiac glycosides were detected in the algal methanolic fractions based on standard methods^21^.

### Antimicrobial activity assay

The bioactivity of the methanolic crude algal fractions was investigated toward four gram negative bacteria: *Vibrio cholerae*,* Pseudomonas aeruginosa*,* Escherichia coli*, and *Aeromonashydrophila*, two gram positive bacteria “*Enterococcus faecalis*and *Staphylococcus aureus*” and one fungal species “*Candida albicans”* employing disc diffusion procedure of Kirby-Bauer procedure^22^. Employing cotton swabs, bacterial cultures containing “150 CFU/ml” were uniformly added to nutrient agar plates. Around 0.75 × 10^6^ fungal spores/ml were aseptically swabbed Czapex-Dox plates. Sterilized discs (6 mm) from Whatman No. 1 filter paper were impregnated with either extract (10 mg/ml) and dried entirely in sterile circumstances subsequently were maintained at 37 °C for 24 h for bacteria and at 28 °C for 48-72 h for fungal forms, correspondingly. DMSO served as a negative control, while the positive control was ciprofloxacin (10 mg/ml). The diameter of clear zone plus the diameter paper disc (mm) was used to calculate antibacterial activity. The average of triplicate analyses is used for all estimated antibacterial outcomes.

### GC-ISO Mass analysis of methanolic algal extract

A GC-ISQ mass spectrometer (Thermo Scientific, Austin, TX, USA) was employed to analyze bioactive complexes present in various methanolic preparations. The analysis was carried out applying a TG–5MS capillary column (30 m × 0.25 mm × 0.25 μm film thickness). The temperature of the oven was initially set at 55 °C, subsequently elevated at a pace of 5 °C/min to 250 °C, sustained for 2 min, and finally rose to 300 °C at 25 °C/min. The injector temperature was set at 270 °C. The carrier gas was helium at a steady flow rate of 1 mL/min. A solvent delay of 4 min was applied, and 1 µL of each diluted sample was automatically introduced in split mode employing the AS3000 Autosampler integrated with GC. In full scan mode, electron ionization (EI) mass spectra were obtained at 70 eV across the m/z interval of 50–650. Compound identification was executed by contrasting retention intervals and mass spectra with entries in the NIST14 and WILEY 09 mass spectral libraries^23^.

### Statistical analysis

All experimental treatments and chemical assays were conducted in independent triplicates ($*n* = 3$). Data are presented as mean ± standard deviation (SD). The findings for diverse biochemical variables were interpreted by one-way ANOVA followed by Duncan’s multiple range test to compare differences between means ($*P* < 0.05$) using SPSS software. Prior to running the ANOVA, the underlying assumptions of data normality and homogeneity of variances were verified and met using the Shapiro-Wilk and Levene’s tests, respectively.

## Results and discussion

### Microalgae Isolated

**Fig. 1:**
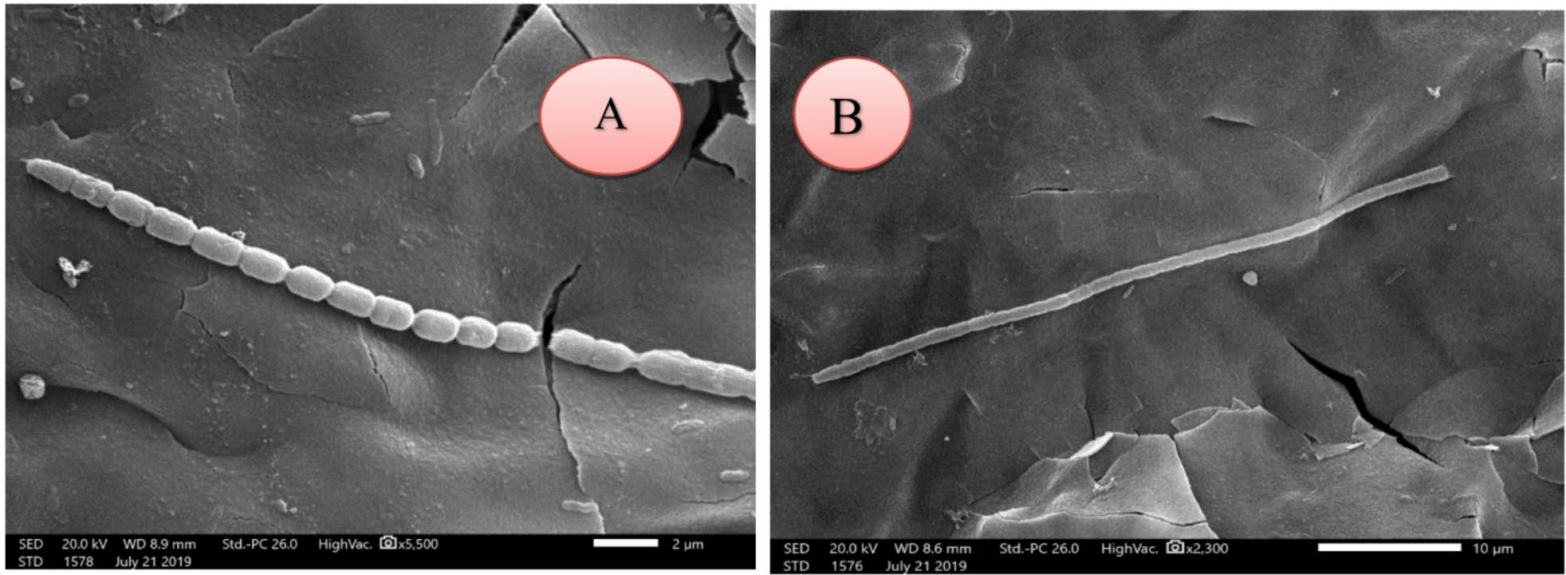
Scanning electron microscope (SEM) images of (**A**) *Oscillatoria acutissima* (**B**) *Oscillatoria simplicissima*.

*Oscillatoria acutissima* and *Oscillatoria simplicissima* were the identified algal strains For taxonomic verification via scanning electron microscopy (SEM), fresh filaments were fixed in a 2.5% glutaraldehyde solution prepared in 0.1 M phosphate-buffered saline (PBS, pH 7.2) for 4 h at 4 °C. The samples were rinsed three times with PBS and then dehydrated through a graded ethanol series (30%, 50%, 70%, 90%, and 100% $v/v$) for 15 min per step. After dehydration, specimens were critical-point dried, attached to aluminum stubs using double-sided conductive carbon tape, and coated with a thin layer of gold using a sputter coater according to standard microalgal preparation protocols (Fig. 1).

The two cyanobacterial isolates were clearly distinguished using distinct morphological markers visible under SEM. *Oscillatoria acutissima* (Fig. 1A) was identified by its thinner trichomes and characteristically attenuated, tapering, and sharply pointed apical cells. In contrast, *Oscillatoria simplicissima* (Fig. 1B) displayed noticeably thicker trichomes ending in straight, non-attenuated, and broadly rounded apical cells. It should be explicitly noted that image B was intentionally captured at a lower magnification than image A. This difference in magnification was intentionally utilized to display the longer, uniform, and non-tapering continuous length profile of the *O. simplicissima* filament, which is a critical diagnostic metric required to accurately separate it from *O. acutissima*.

**Fig. 2:**
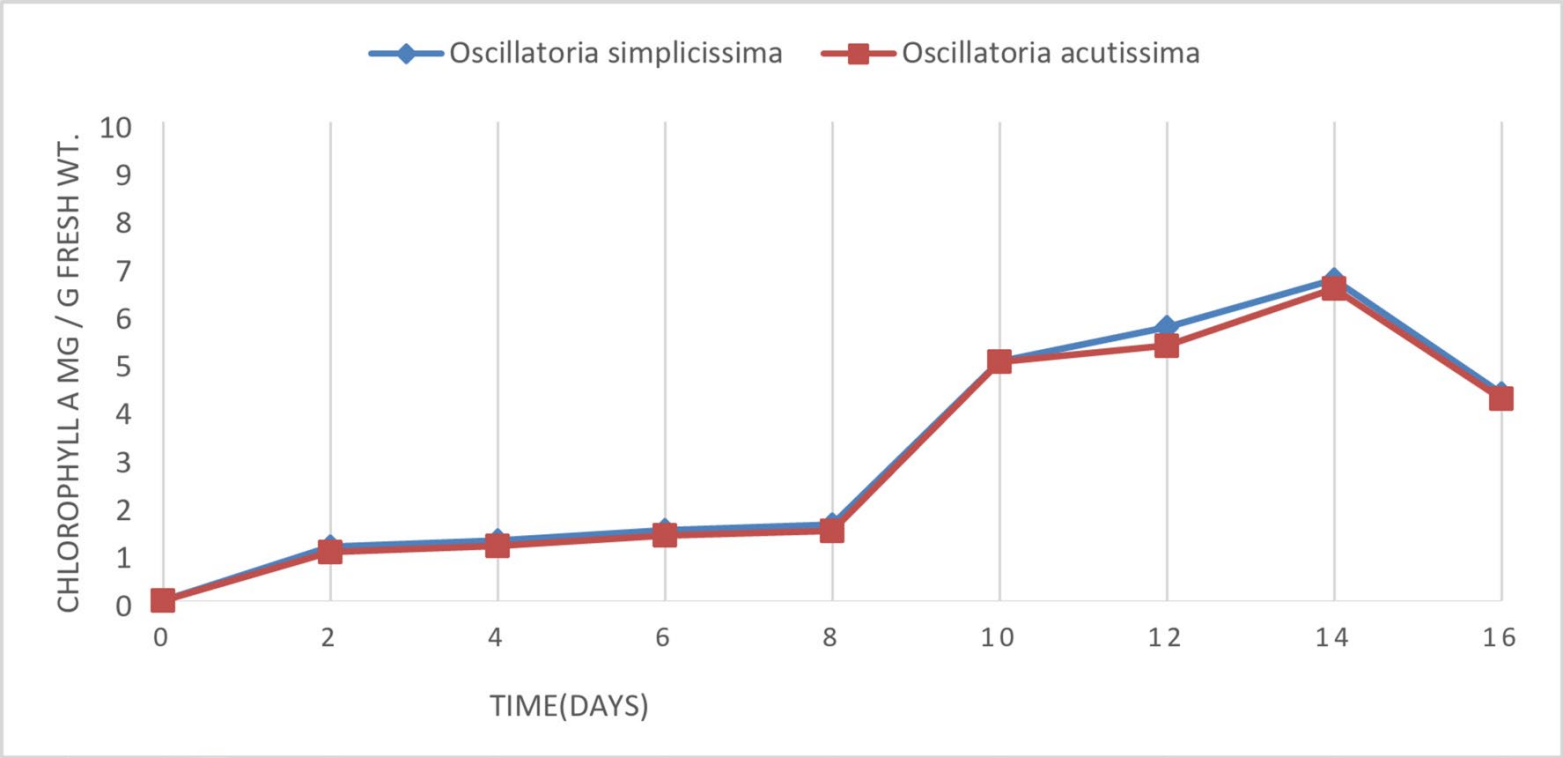
The growth *of Oscillatoria acutissima* and *Oscillatoria simplicissima* assessed as chlorophyll (a) mg/g fresh wt.

At a temperature of 30 ± 2 °C, pH of 8, and light intensity of 3000 lx, the algal strains were collected at the 14th day of their exponential phase of growth, which corresponds to Oscillatoria *acutissima* and *Oscillatoria simplicissima* (Fig. 2). The majority of research on algal biochemical synthesis and analysis was done during the stationary phase of growth^25^.

The growth of *Oscillatoria acutissima* and *Oscillatoria simplicissima* assessed as chlorophyll (a) mg/g fresh wt. Data points represent the means of three independent biological replicates ($*n* = 3$), and error bars indicate the standard deviation (± SD).

### Qualitative analysis of phytochemicals

Marine microalgae are one of the greatest nature providers of cosmetic industry, medicines and food owing to their phytochemical compositions. Likewise displayed in Table 1, the initial phytochemical components of the algal methanolic extracts revealed differences among algal species. These differences may be associated with algal species, culture settings, extraction procedure and the employed solvent^26^. Phenol, phytosterols, terpenes, tannins, flavonoids, and coumarins are found in all assessed. These secondary metabolites exhibit a broad interval of antimicrobial^27^. Saponin is detected in *C. marina*,* O. acutissimaand S. platinesiswhich* it is utilized in the feeding of the animals. Glycosides may be employed in the therapy of some heart disorders, for example, cardiac arrhythmia^28^, It was noted in the prior 3 species.

**Table 1:** Qualitative assessment of the phytochemical constituents of the identified algae.

Spp. Phytochemicals	Cyanobacteria spp.	
	Oscillatoria simplicissima	Oscillatoria acutissima
Phenolics	**+**	**++**
Flavonoids	**+**	**+**
Tannins	**+**	**++**
Saponins	**-**	**+**
Triterpenes	**+**	**+**
Anthraquinones	**-**	**-**
Coumarins	**+**	**+**
Cardiac glycosides	**+**	**-**

A high (++), moderate (+) or absent (-) according to the strength of the color developed throughout the reaction.

### Antimicrobial activity

Cyanobacteria species are a sustainable origin of new active metabolites with various biological activities, including antioxidants, antifungal, antibacterial, and antitumor characteristics^29^. The crude methanolic extracts of the grown algae forms are evaluated toward diverse pathogens and the findings are displayed in Table 2. The extracted methanol of *O. simplicissima* (11–17 mm), and *O. acutissima* (14–20 mm). The assessed activity of most examined algae was more antimicrobial activity relative to *Ph. fragile* (12–13 mm)^30^. Similarly, Srinivas akumar and Rajashekhar (2009) who demonstrated *C. marina* methanolic extract registered greatest inhibitory activity in an investigation toward *P. aeruginosa*,* P. fluorescensand S. typhi.* The fluctuation of gene expression combined signal transduction. Algal fatty acids, specifically, may trigger peroxidation and hinder the synthesis of bacterial fatty acids^31^. Additionally, fatty acid methyl esters and free fatty acids can disrupt microbial cells by interacting with their membranes, leading to leakage of cellular molecules, reduced nutrient absorption, or inhibition of respiration^32^. These compounds likely function either independently or synergistically. Methanol extracts from all tested algae exhibited antifungal effects against C. albicans, with the largest inhibition zone (20 mm) recorded for Oscillatoria simplicissima. This finding aligns with previous reports^33^. Notably, marine microalgae of the genus Oscillatoria demonstrated stronger antifungal activity compared to their antibacterial effects^34^.

**Table 2:** Antimicrobial activity of the algal methanolic extract toward various pathogenic microorganisms.

Diameter of inhibition zone (mm)								
Spp. Standard antibiotics		Gram (+ V) bacteria		Gram (-V) bacteria			Fungal sp.	
		S. aureus	E. faecalis	*P*. aeruginos]	A. hydrophila	V. cholera	E. coli	C. albicans
**Ciprofloxacin (+ ve)**		**20 ± 0.01**	**22 ± 0.02**	**22 ± 0.02**	**22 ± 0.02**	**22 ± 0.02**	**22 ± 0.02**	**20 ± 0.01**
**DMSO (-v)**		**-**	**-**	**-**	**-**	**-**	**-**	**-**
	***O. simplicissima***	**12 ± 0.3**	**11 ± 0.3**	**14 ± 0.2**	**17 ± 0.2**	**13 ± 0.2**	**13 ± 0.2**	**11 ± 0.2**
	***O. acutissima***	**16 ± 0.2**	**15 ± 0.2**	**14 ± 0.2**	**18 ± 0.3**	**17 ± 0.3**	**17 ± 3**	**20 ± 0.3**

### Gas chromatography-mass spectrometry (GC-MS)

**Fig. 3:**
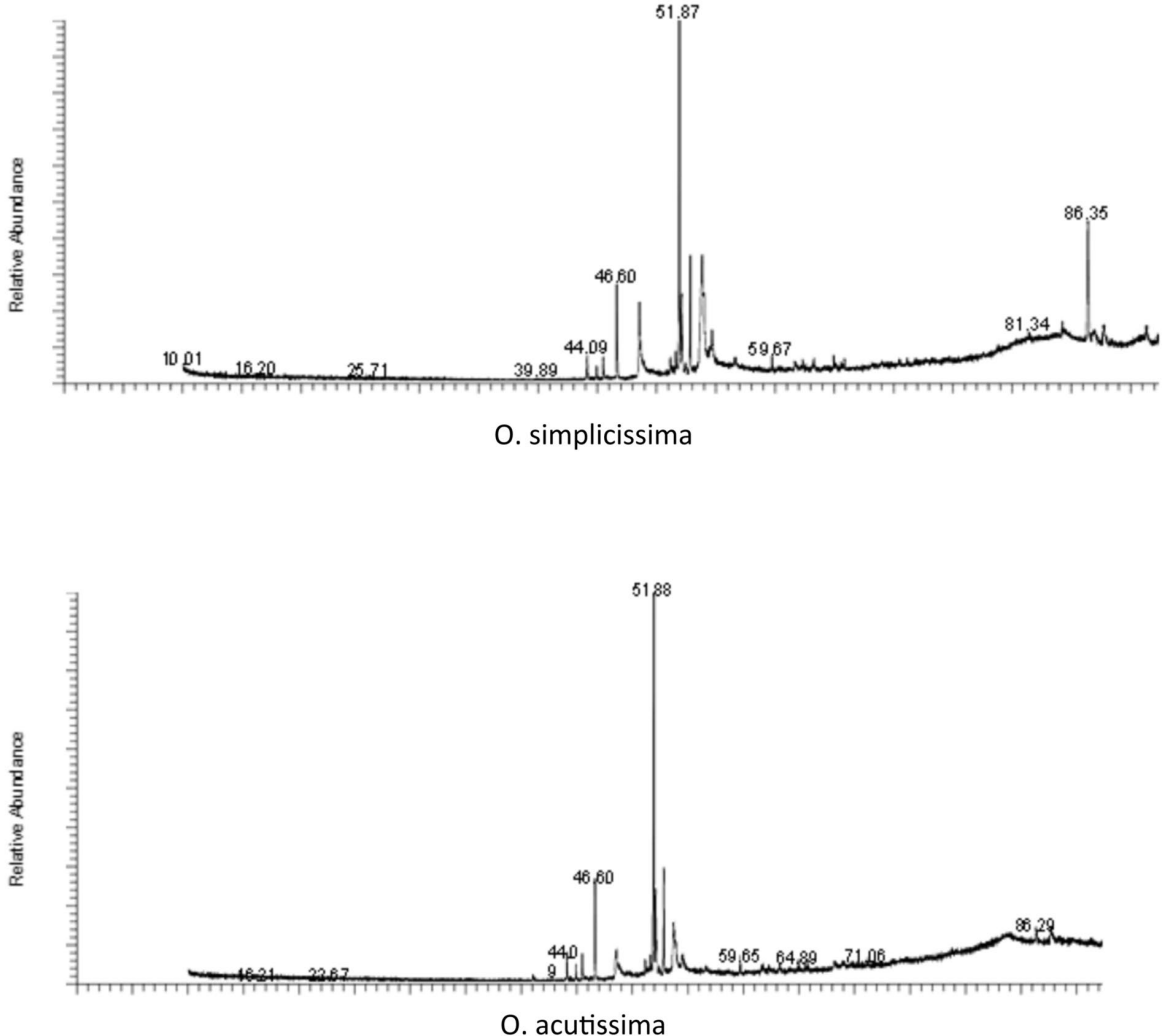
Chart of GC/MS chromatograms and results of *O. simplicissima* and O. acutissima .

*Oscillatoria simplicissima* and *Oscillatoria acutissima* , two closely related species, were analyzed by GC-MS on methanol extracts (Fig. 3). Samples *O.*  *simplicissima* and *O. acutissima* exhibited a chemical link as they share most of the main compounds, such as 11-Octadecenoic acid, oleic acid and their methyl esters.

In (Table 3), *O. acutissima* had a higher total FAMEs content (~ 63%) with more unsaturation, *O.  simplicissima* has more hydrocarbons, notably heptacosane (9.95%), this indicates a waxy or less processed matrix. The major compound in both *Oscillatoria* species is 11-Octadecenoic acid, methyl ester, its content is 25.54%, 34.90% in *O.  simplicissima* and *O. acutissima*, respectively. It was identified previously in another species, freshwater Microalga *Oscillatoria princeps* contributing its antimicrobial and cytotoxic potentials^35^. Oleic acid has the second proportion in *O.  simplicissima* greater than *O. acutissima*, it postulated that lipids impair microbes by causing the cellular membrane to rupture^36^. In the other side, a phytochemical study was recorded in *Oscillatoria* sp. with different acids including methyl stearate that occupied the second area percentage in   *O. acutissima* much higher than its content in *O.  simplicissima*. Methyl Stearate with other components had antimicrobial and antioxidant capacities^37^. Many Shared compounds are reported in both species as Neophytadiene, Methyl Stearate, Oleic Acid, 3,7,11,15-Tetramethyl-2-hexadecen-1-ol, n-Hexadecanoic acid, methyl esters of many acids as 13-Docosenoic acid, Docosanoic acid, Eicosanoic acid, 9,12-Octadecadienoic acid, 11-Octadecenoic acid, 9-Octadecenoic acid and 9-Hexadecenoic acid. 9,12-Octadecadienoic acid, Phenol 2,2’-methylenebis[6-(1,1-dimethylethyl)-4-methyl-13-Octadecenoic acid, Hexadecanoic acid, 2,3 dihydroxypropyl Ester, 9-Octadecenoic acid, 1-octadecanal, Heptacosane and 2-hydroxy-3-[(9e)-9 octadecenoyloxy] propyl (9e)-9-octadecenoate.

Also, many compounds were present in one species and absent in the other one. Sex compounds; 7-methyl Heptadecane, 9-Eicosyne, 17-Octadecynoic acid, 9-Octadecenoic acid,1,2,3-propanetriyl ester, 1,2-Benzenedicarboxylic acid, 2-(acetyloxy)-1-[(acetyloxy) methyl] ethyl ester, and 9,12,15-Octadecatrienoic acid, are absent in *O.  simplicissima*. In other side, another different sex compounds; Vaccenic acid, Palmitic acid-2(tetradecyloxy) ethyl ester, Octadecanoic acid, 4-H-1-benzopyran-4-one-2-(3,4-dihydroxyphenyl)-6,8-di-α-D-glucopyranosyl-5,7-dihydroxy, Ethyl iso-allocholate and Trilinolein are absent in  *O. acutissima .* In conclusion, both *Oscillatoria simplicissima* and *Oscillatoria acutissima* are rich in oleic and stearic acid derivatives. *O. acutissima* shows enhanced unsaturation and ester content, suggesting it may originate from a more metabolically active source, while *O.  simplicissima *exhibits higher hydrocarbon content and unique components, implying a more waxy or less processed lipid profile., their algal methanol extract was very efficient toward bacterial and fungal strains^38,39^. Natural products function and needs have been further enhanced by their function as repositories of novel structural templates, which are abundant in nature as byproducts of the plants’ secondary metabolism^40,]^

**Table 3:**
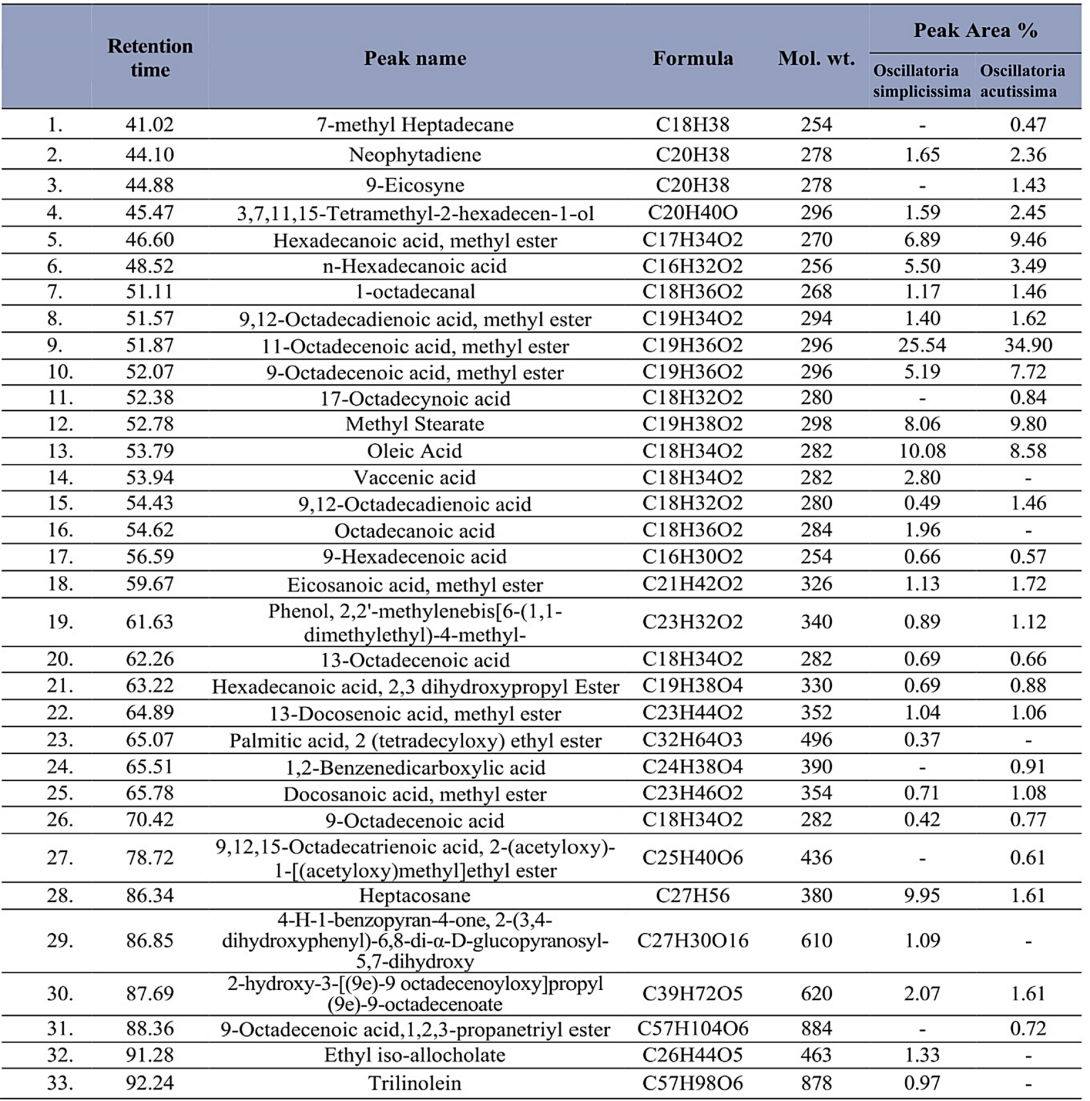
GC/MS identified components of ***O. simplicissima*** **and** ***O. acutissima ***.

## Conclusion

The present study demonstrated the marine cyanobacteria species methanolic extracts contained interesting phytochemical profile such as Phenol, coumarins, phytosterols, terpenes, tannins, and flavonoids. These compounds act as a source of functional ingredient that can be developed these compounds as potent antimicrobial compounds. Consequently, further research is needed to identify the appropriate culture conditions to achieve elevated concentrations of bioactive contents. Beside to using genome editing tools may be increase the output of marine cyanobacteria and their bioactive complexes. Additional fractionation and purification of algal extract for delivering several active, safe and renewable drugs.

## Data Availability

All data generated or analysed during this study are included in this published article.
